# Valorization of sugarcane bagasse by chemical pretreatment and enzyme mediated deconstruction

**DOI:** 10.1038/s41598-019-52347-7

**Published:** 2019-11-04

**Authors:** Vihang S. Thite, Anuradha S. Nerurkar

**Affiliations:** Department of Microbiology and Biotechnology Centre, Faculty of Science, The Maharaja Sayajirao University of Baroda, Vadodara, Gujarat 390002 India

**Keywords:** Biocatalysis, Enzymes, Chemical biology, Microbiology

## Abstract

After chemical pretreatment, improved amenability of agrowaste biomass for enzymatic saccharification needs an understanding of the effect exerted by pretreatments on biomass for enzymatic deconstruction. In present studies, NaOH, NH_4_OH and H_2_SO_4_ pretreatments effectively changed visible morphology imparting distinct fibrous appearance to sugarcane bagasse (SCB). Filtrate analysis after NaOH, NH_4_OH and H_2_SO_4_ pretreatments yielded release of soluble reducing sugars (SRS) in range of ~0.17–0.44%, ~0.38–0.75% and ~2.9–8.4% respectively. Gravimetric analysis of pretreated SCB (PSCB) biomass also revealed dry weight loss in range of ~25.8–44.8%, ~11.1–16.0% and ~28.3–38.0% by the three pretreatments in the same order. Release of soluble components other than SRS, majorly reported to be soluble lignins, were observed highest for NaOH followed by H_2_SO_4_ and NH_4_OH pretreatments. Decrease or absence of peaks attributed to lignin and loosened fibrous appearance of biomass during FTIR and SEM studies respectively further corroborated with our observations of lignin removal. Application of commercial cellulase increased raw SCB saccharification from 1.93% to 38.84%, 25.56% and 9.61% after NaOH, H_2_SO_4_ and NH_4_OH pretreatments. Structural changes brought by cell wall degrading enzymes were first time shown visually confirming the cell wall disintegration under brightfield, darkfield and fluorescence microscopy. The microscopic evidence and saccharification results proved that the chemical treatment valorized the SCB by making it amenable for enzymatic saccharification.

## Introduction

After consuming sugarcane for sugar production, sugar factories yield a huge amount of solid byproduct known as sugarcane bagasse (SCB), i.e., a retained fibrous material after crushing and squeezing the sugarcane^1^. After removal of soluble sugars, SCB has been reported to possess approximately 41.4% glucan, 28.2% xylan, 1.3% galactan and 23.6% lignin as structural polymers in composition^2^. Besides cogeneration, biotechnological potentials of SCB have been explored for production of several chemicals, alkaloids, metabolites and enzymes during fermentation process, in pulp and paper industries and for production of bioethanol, methane and butanol in biorefinery^3–6^.

Biorefineries depend on the two sequential fundamental steps of biomass saccharification followed by fermentation of the released sugars. The saccharifying enzymes that act on different plant polysaccharides are categorized as core and accessory enzymes where cellulases and xylano-pectinolytic enzymes belong respectively. Though the depolymerization and saccharification of plant cell wall necessitates action of a cocktail of enzymes comprising of cellulases with xylano-pectinolytic enzymes, this involvement alone is insufficient for obtaining best enzymatic saccharification. Such inefficiencies can be attributed to recalcitrant nature of plant cell wall, strong crystalline cellulose arrangements, unproductive binding of enzymes to several structural components, enzyme inactivation over the incubation time and inhibition due to end-product accumulation^1,4^. Recalcitrance of plant cell wall is a major factor that restricts biomass accessibility to plant cell wall degrading (PCWD) enzymes and can be credited to the complex cell wall matrix formed by strong interactions among cellulose, hemicellulose, pectin and lignin. Thus, the biorefinery process demands partial fractionation of cell wall matrix and their utmost amenability towards enzymatic depolymerization^5^. This can be achieved through applying different pretreatments to agro waste biomass before the enzymatic saccharification. Due to the diverse nature of biomass feedstock, it is tough to develop a single pretreatment process which is applicable to all. In literature different combinations of several pretreatment strategies based on the pretreatment agents, forces and energy used in various types of agro-waste biomass have been reported^7–9^. Mechanical pretreatments by comminution, milling or extrusion reduce the biomass particle size, ease the material handling, increase surface to volume ratio. Although mechanical processes yield better substrate accessibility during saccharification process, the complex matrix of biomass becomes limiting factor and requires the assistance of chemical, physico-chemical or biological pretreatments^10^. Chemical pretreatments involve use of acid, alkali, liquid ammonia, organic solvent. When the physical parameters, like compression and expansion of volume, pressure, heat, microwave and sonication are invovled, the methods are referred to as physico-chemical pretreatments. Different chemical reagent loadings along with several modifications in physical factors like residence or holding time, temperature and pressure conditions during these pretreatments have been studied^10–13^. Such combinations variedly affect biomass by altering the lignocellulosic structural components and improve the accessibility of glycosidic linkages to the PCWD enzymes and enhances their enzymatic saccharification activity, i.e., biomass deconstruction.

Improved % glucan conversion or enzymatic saccharification after acid pretreatments with acid have been reported for sugarcane bagasse (~30% glucan conversion), wheat straw (~ 60% saccharification) and corn stover (~60–80% glucan conversion) as pretreatment rendered cellulosic polymers moderately accessible by partial removal of hemicellulose and acid soluble lignin fractions^14–16^. Improved enzymatic saccharification of rice straw (~55% saccharification from NaOH pretreated, ~60–75% glucan conversion from AFEX pretreated), and SCB (~60–70% saccharification from NaOH PSCB, ~40–60% saccharification from AFEX-PSCB) have been reported after these pretreatments as they efficiently removed lignin content without altering polysaccharides^16–19^. The reported studies used cocktails of commercial crude preparations of cellulases like Spezyme CP, Novozyme 188, Primafast 200, Accellerase 1500, CTec2 along with hemicellulases like Multifect xylanase and Multifect pectinase. In spite of investing efforts and funds for past few decades, the enzymatic saccharification process is yet to be optimized for maximum efficiency at the commercial plant level^20,21^.

Several features have been reported to be mandatory for an efficient pretreatment. It should produce very negligible amounts of soluble sugar and lignin degradation products along with high recovery of carbohydrates as solid biomass. It should impart high digestibility of the carbohydrates during subsequent enzymatic hydrolysis step and the carbohydrate released should be further useful for fermentation without detoxification. Process should have low energy requirement, operational cost and capital investment^11^.

Available reports have depicted the chemical and structural changes in biomass using different techniques like SEM, FTIR, X-ray diffraction etc., and biomass accessibility in terms of glucan conversion or saccharification using diverse enzymes. Light and fluorescence based microscopic direct visual evidence of cellulase action on the plant cells from pretreated biomass are rare. The Primafast 200 is commercial cellulase with whole cellulase spectrum which have been explored as core enzymes for raw agrowaste biomass saccharification along with crude accessory enzymes^22–24^.

In this context, the aim of the present work was to study the effects of pretreatments on the structure of biomass and how these changes make its structural polysaccharide components accessible to the PCWD enzymes. For this purpose, following objectives were taken up. (I) Pretreatment of SCB biomass, (II) Biochemical and HPLC based analysis of pretreatment filtrate for released soluble sugars, (III) Characterization of pretreated biomass through Gravimetric, FTIR and SEM analyses for structural and chemical alterations, (IV) Assessment of enhanced accessibility of structural polysaccharide to PCWD enzymes through biochemical and microscopic method.

## Results and Discussion

In present studies, the SCB biomass was subjected to mechanical comminution followed by chemical pretreatments i.e., alkali (NaOH), acid (H_2_SO_4_) and liquid ammonia (NH_4_OH) for improving enzymatic digestibility (Strategy outlined in Supplementary Fig. 1). Keeping the physical parameters i.e., temperature, holding time and pressure constant, effect of different biomass loadings and chemical loading were discussed in terms of morphological changes, released SRS, released solubles other than reducing sugars, weight loss and enzymatic digestibility of biomass which subsequently help in concluding the pretreatment efficiency.

### Changes in visible morphology of sugarcane biomass

In present studies, changed visible morphology of SCB biomass, after application of chemical pretreatments, have been depicted in the Supplementary Fig. 2. When compared to raw untreated SCB biomass control, distinct separation of fibrous and stiff vascular bundles from the shattered, fluffy and soft parenchymatous pith mass were clearly visible from pretreated SCB biomass (Supplementary Fig. 2a–d). Increased transparency of the soft pith region from the vascular bundles imparted fibrous appearance as evidently seen from the cuboids of sugarcane stem subjected to NaOH, NH_4_OH and H_2_SO_4_ pretreatments (Supplementary Fig. 2e). Such changes can be attributed to the alterations in the structural components of cell wall matrix brought by the applied pretreatments. While ammonia and alkali pretreatments have been reported to solubilize major to most lignin fractions respectively, acid pretreatment has been reported to dissolve only acid soluble fraction of lignin along with hemicelluloses through different chemical reactions during chemical or physico-chemical pretreatments^8,9^. This can make biomass more porous to overcome the hurdles in saccharification. Moreover, NH_4_OH as well as NaOH pretreatments imparted pale yellow to yellow coloration respectively (Supplementary Fig. 2b,c), and acid pretreatment imparted the pink-red coloration to biomass (Supplementary Fig. 2d). These colorations can be attributed to the interactions of the polysaccharide components with the chemical reagents used for pretreatments. Increased yellow colour of the biomass has been reported from SCB after treatment with NaOH, Ca(OH)_2_ and NH_4_OH^25^. Whereas, mineral acid has reacted with polysaccharides (majorly cellulose) to form furfurals and with lignin which imparted the pink-red colour to the biomass^26^.

### Release of soluble reducing sugars (SRS) in pretreatment filtrate

**Figure 1:**
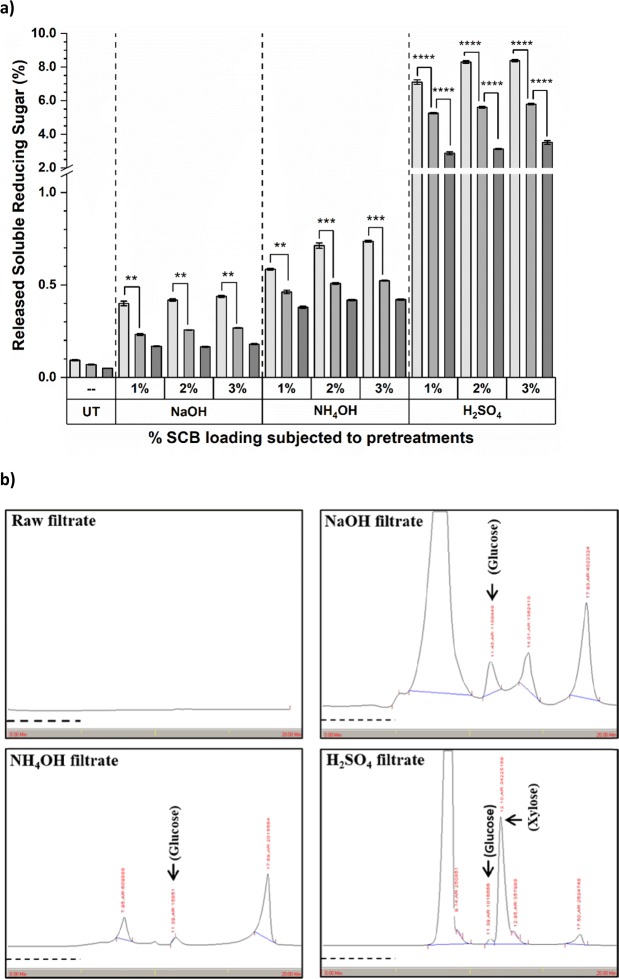
Release of soluble components in pretreatment filtrate from raw and PSCB. (**a**) Release of % SRS in pretreatment filtrate from raw untreated control, NaOH, NH_4_OH and H_2_SO_4_ pretreatments; Significance difference between 1.25% (), 2.5% () and 5.0% () SCB biomass loading for pretreatments is given as *p < 0.05, **p < 0.01, ***p < 0.001 and ****p < 0.0001 for Sidek test-Two-way ANOVA analysis; Values presented are Mean ± Standard Errors of the Mean (SEM), for n = 3. (**b**) Representative chromatographs of HPLC analysis for pretreatment filtrates of 5% (w/v) loading of SCB for Raw untreated control, 3% NaOH, 3% NH_4_OH and 3% H_2_SO_4_ pretreatment; RT: Glucose ~11.4 min, Xylose ~12.1 min; Dashed line () at X-axis indicates the time span of 5 min on chromatogram; n = 3.

Biochemical estimations unveiled release of SRS less than 0.1% from the filtrate of untreated SCB biomass. The value substantially increased after the chemical pretreatments (Fig. 1a). NaOH pretreatment released ~ 0.2–0.5% SRS which further increased to the range of ~ 0.4–0.8% for NH_4_OH pretreatment and reached to maximum in range of ~ 4.0–8.0% after H_2_SO_4_ pretreatment. Among all, 1.25% SCB loading exhibited maximum amount of released SRS. Two-Way ANOVA analysis suggested that, the difference in released SRS for 1.25% and 2.5% SCB loading was significant for all pretreatments but that between 2.5% and 5.0% was significant only for H_2_SO_4_ pretreatments (*p* < 0.0001). During HPLC analysis, peak corresponding to glucose (RT 11.4 min) and xylose (RT 12.1 min) monomers were completely absent from filtrate of raw untreated SCB (Fig. 1b). A peak corresponding to glucose was detected from NaOH, NH_4_OH and H_2_SO_4_ pretreatment filtrate, the last one being the notably large among all three. Whereas, a huge peak corresponding to xylose was observed only in H_2_SO_4_ pretreatment filtrate, that too with several fold more undercover area than glucose. These observations suggested that NaOH and NH_4_OH pretreatments negligibly hydrolyzed cellulose fractions without affecting hemicellulosic fraction. On contrary, acid pretreatments majorly hydrolyzed hemicellulose from the cell wall matrix to xylose monomers forming a major fraction of released SRS from H_2_SO_4_ pretreatment filtrate making the glucose minor component. Our results corroborated with the literature, where unaltered cellulose content from hydrothermally, alkali and acid pretreated biomass and decreased hemicellulose content from acid pretreated biomass only have been reported from eucalyptus, SCB and straw^13^. In studies with different combinations of SCB and acid loadings during pretreatments, ~ 90–94% loss of hemicellulose content and relative increase in cellulose content have been reported^27^. Further, peaks observed at longer RT after glucose and/or xylose from chemical pretreatment filtrates pointed out the presence of other extractives which involves water, acid and alkali soluble components besides these reducing sugars^13^.

### Loss of dry weight from SCB biomass during pretreatment

**Figure 2:**
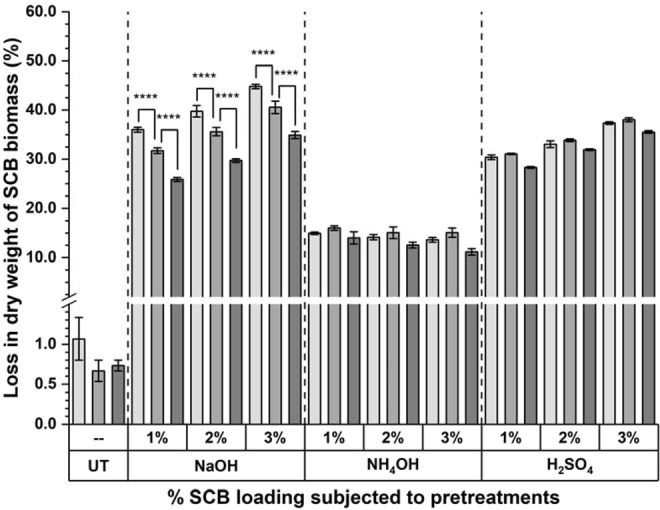
Loss in dry weight (%) from raw and pretreated biomass. % Loss in dry weight from raw untreated control, NaOH, NH_4_OH and H_2_SO_4_ pretreatments; Significance difference between 1.25% (), 2.5% () and 5.0% () SCB biomass loading for pretreatments is given as *p < 0.05, **p < 0.01, ***p < 0.001 and ****p < 0.0001 for Sidek test-Two-way ANOVA analysis; Values presented are Mean ± Standard Errors of the Mean (SEM), for n = 3.

Gravimetric estimations, revealed less than 1% loss in dry weight from raw untreated SCB biomass. The dry weight loss markedly increased after subjecting biomass to the chemical pretreatments (Fig. 2). This loss was maximum and in range of ~26.8–44.8% for NaOH pretreatment and decreased to ~25.3–38.0% for H_2_SO_4_ pretreatment. Whereas, ~11.1–16.0% loss was observed for NH_4_OH pretreatments. Two-Way ANOVA suggested that the difference observed for the % weight loss was significant for each of SCB loading in all three NaOH pretreatments (*p* < 0.0001). These results reasserted the reported observations of 15.4, 49.3 and 49.1% loss in dry weight of SCB due to hydrothermal, acidic and alkaline pretreatments respectively and along with a similar pattern of weight loss for eucalyptus and sugarcane straw biomass^13^.

### Release of soluble components other than SRS from SCB biomass during pretreatment

Three different biomass loadings and three different chemical loadings generated a matrix of nine combinations for each pretreatment which was used to prepare contour plot for % released SRS and % dry weight for each chemical pretreatment and compared (Supplementary Fig. 3a–f). Comparing the results of pretreatment filtrate biochemical estimations with gravimetric analysis of PSCB suggested a huge difference in the values of % SRS and loss in dry weight. On combining these observations with the several peaks observed in HPLC chromatograms, it can be inferred that besides the released SRS, there must be other extractive components which were solubilized in alkali, ammonia or acid from the biomass during these pretreatments. These differences are calculated in terms of the amount of solubles components released other than SRS during pretreatments and are depicted in Table 1. Only ~0.71–0.75% other soluble components were released in water from raw SCB biomass. Which significantly increased to the values falling in range of ~25.6–44.3%, ~23.3–32.2% and ~10.7–15.5% after NaOH, H_2_SO_4_ and NH_4_OH pretreatment respectively (ANOVA at *p* < 0.001). NaOH released highest solubles at 1.25% SCB loading, but NH_4_OH and H_2_SO_4_ released highest solubles at 2.5% SCB loadings. In literature, the major proportion of such solubles in filtrate of NaOH and H_2_SO_4_ pretreatments are reported to be alkali soluble or acid soluble fraction of lignin^28^. Moreover, peaks observed at the extended RT (longer RT then glucose and xylose) can be attributed to such soluble components and their longer retention time was possibly due to the strong hydrophobic interactions between column matrix and the compounds which are possibly released by lignin solubilization. Similarly, increase in fraction of cellulose content with minor decrease in hemicellulose content from recovered dry SCB biomass after NaOH pretreatment have been reported^29^. Though, these observations were reported for biomass, their extrapolation to the filtrates and their correlation with our observations of present studies infers that NaOH and NH_4_OH treatments affected cellulose and hemicellulose negligibly but H_2_SO_4_ dissolved major hemicellulose content. Thus, the results suggested that H_2_SO_4_ pretreatment removed a major part of hemicellulose and an acid soluble fraction of lignin, keeping the cellulosic components and insoluble lignin unaltered. But the alkali and ammonia pretreatments retained the cellulose and hemicellulose both with removal of major fraction of lignin.

**Table 1:** % Solubles released from biomass other than SRS during different pretreatments.

SCB (% w/v)	Pretreatment									
	Raw	NaOH (% w/v)			NH_4_OH (% v/v)			H_2_SO_4_ (% v/v)		
		1	2	3	1	2	3	1	2	3
1.25	0.71	**35.60**	**39.32**	**44.36**	14.35	13.42	12.86	23.30	24.77	28.96
2.50	0.73	31.50	35.34	40.27	**15.54**	**14.56**	**14.54**	**25.81**	**28.80**	**32.20**
5.00	**0.75**	25.70	29.57	34.75	13.62	12.12	10.71	25.46	28.27	32.02

Values in bold indicates the highest values; Values presented are Mean for n = 3.

### FTIR analysis of raw and pretreated SCB biomass

**Figure 3:**
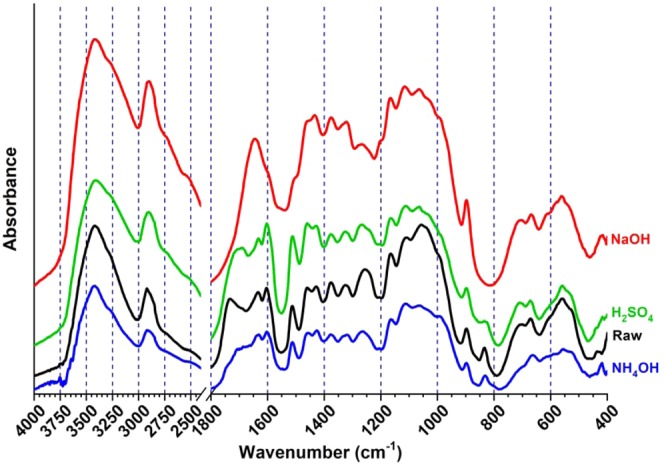
FTIR spectra of raw and PSCB biomass fragments. FTIR spectra of representing chemical changes in untreated (Black, ), NaOH (Red, ), NH_4_OH (Blue, ) and H_2_SO_4_ (Green, )pretreated sugarcane bagasse (SCB) biomass solids at wavenumbers of 400–4000 cm^−1^; Y-axis is presented as an offset.

The absorption peaks or bands, attributed to functional chemical groups or the chemical bonds present from the lignocellulosic biomass, are mainly clustered in two different range of wavenumbers, i.e., 3750–2750 cm^−1^ and 1800–800 cm^−1^ for FTIR analysis (Fig. 3). The strong and broad absorbance peak observed at the range of 3450–3300 cm^−1^ and 2950–2820 cm^−1^ were due to stretching exhibited by O-H bond of -OH groups and symmetrical or asymmetrical -C-H stretching vibrations for -CH_3_, -CH_2_ or -CH groups present in lignocellulosic fractions. Peaks at wavenumbers 1734, 1630, 1600, 1510, 1325, 1270, 1060 and 833 cm^−1^, attributed to attributed to chemical bonds and structures present in aromatic lignin components, were significantly affected by pretreatments when compared with raw untreated biomass. Decrease or complete disappearance of peak 1734 cm^−1^ from these spectra suggested efficient breakdown of cross-linkages between acetyl group of hemicellulose and lignin components which probably led to the separation of the lignin components from the cellulose-hemicellulosic complex matrix from all three types of PSCB. Peaks at 1734, 1510, 1060, 1030 and 833 cm^−1^ were absent from NaOH PSCB spectrum; peaks at 1630, 1600, 1510, 1325, 1270, 1070, 1030 cm^−1^ wavenumbers decreased from NH_4_OH PSCB spectrum. Whereas, peaks at 1734, 1630, 1325, 1270, 1060 and 833 cm^−1^ were decreased in spectrum of H_2_SO_4_ PSCB suggested removal or decrease of lignin. Peak at 833 cm^−1^ was attributed to out of plane C-H deformations of aromatic ring, decrease and absence of which also pointed towards decrease and absence of aromatic lignin from NH_4_OH, H_2_SO_4_ and NaOH pretreatments. Similar effects of delignification have been observed for AFEX treated rice straw and NaOH treated SCB^25,30^. In literature, similar changes at these wavenumbers have been reported after biological, acid, ammonia or alkali mediated delignification process in literature^29–33^. The peaks at 1430, 1376, 1320, 1160, 1120 1043, 997 and 897 cm^−1^ wavenumbers are characteristic adsorption bands attributed to cellulose and/or hemicellulose^34^. After NaOH and NH_4_OH pretreatments presence of these peaks suggested that cellulosic and hemicellulosic components from PSCB biomass were not drastically affected during these pretreatments. But the peaks at 1043 and 997 cm^−1^ wavenumbers, which are attributed to hemicellulose characteristics, were found to decrease after H_2_SO_4_ pretreatments. In literature also, decreased peaks for hemicellulose characteristics have been reported from acid pretreated biomass^35^. FTIR analysis supports our earlier observations of released solubles other than reducing sugars stated in present studies that the alkali, ammonia pretreatments remove moderate to huge fraction of solubles like lignin and acid pretreatments remove the huge fraction of lignin and hemicellulose content.

### Scanning electron microscopy of raw and pretreated SCB biomass

**Figure 4:**
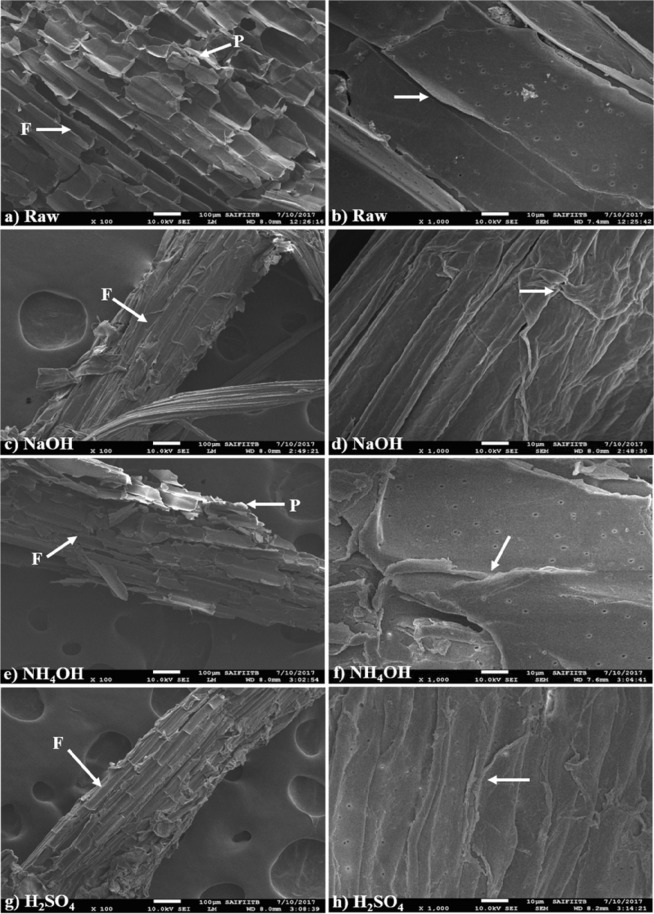
Scanning electron micrographs of raw SCB and PSCB biomass fragments. (**a**,**b**) Raw untreated; (**c**,**d**) NaOH treated; (**e**,**f**) NH_4_OH treated; (**g**,**h**) H_2_SO_4_ treated SCB biomass fragments; Images a, c, e and g were captured at 100X magnification (white bar represents scale of 100 µm); Images (**b**,**d**,**f**,**h**) were captured at 1000X magnification (white bar represents scale of 10 µm).

Effects of pretreatments on fibrous vascular bundles and parenchymatous pith were evidently observed in SEM analysis of raw and PSCB (Fig. 4). In case of raw untreated SCB biomass, fibrous structures (labelled as F) comprised parallel strips of vascular bundle components and are surrounded by soft, fragile pith cells with flaky appearance (labelled as P) as seen from the micrographs (Fig. 4a). And further enhanced magnification revealed the sooth and intact edges of the cell walls from untreated raw biomass along with presence of small pits or pores on the wall that connect the neighboring cells (Fig. 4b). Comparable observations for raw SCB biomass morphology have been reported^16,36^. After pretreatments, separation of fibrous vascular bundles from medullar pith was earmarked in all three cases (Fig. 4c–h). At higher magnification the pretreated samples exhibited enhanced roughness and unevenness on the surface of cell wall as well as increased sharpness of cell wall edges indicated with arrows after all three pretreatments (Fig. 4d,f,h). NaOH and H_2_SO_4_ exhibited drastic changes in morphology of PSCB as it separated the vascular bundles from the pith resulting in the more fibrous features (Fig. 4c,d,g–h). Such increased fibrous appearance have been reported from SCB after alkali and acid pretreatments^16,26,37^. This might have occurred due to removal of soluble lignin fraction from the cells surrounding the vascular bundles. The structural morphology of parenchymatous pith region for NH_4_OH PSCB was majorly affected and enhanced deformations in the cell walls of pith regions gave it scaly appearance (Fig. 3e,f).

Thus, morphological, biochemical and structural analysis of PSCB suggested that these pretreatments have brought several changes in structures of biomass by altering its lignin and/or hemicellulosic components and effectiveness of which can be studied through accessibility analysis of the biomass polysaccharide components to their respective saccharifying enzymes.

### Studies on amenability of the pretreated biomass to enzymatic saccharification

**Figure 5:**
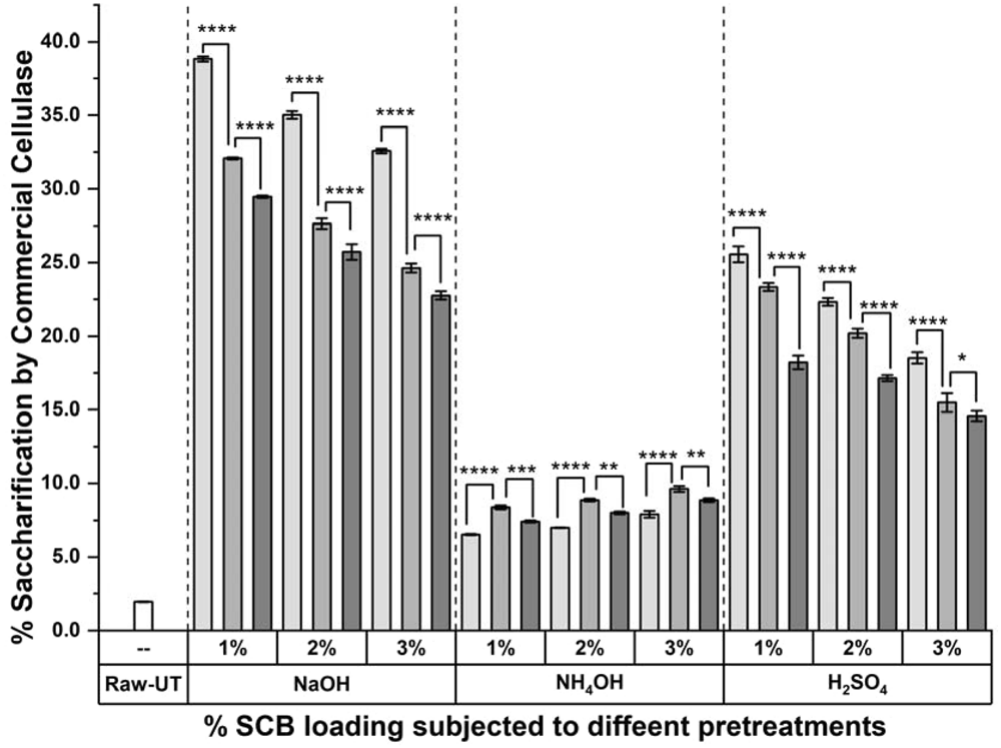
Digestibility of cellulose from raw and PSCB by commercial cellulase. % Saccharification of raw and PSCB exhibited by commercial cellulase.; Significance difference between 1.25% (), 2.5% () and 5.0% () SCB biomass loading for pretreatments is given as *p < 0.05, **p < 0.01, ***p < 0.001 and ****p < 0.0001 for Sidek test-Two-way ANOVA analysis; Columns and Error bars represents Mean and Standard Error of Mean (SEM) respectively for n = 3.

Besides the physico-chemical properties of enzymes and environmental conditions, accessibility of the cell wall polysaccharides to their depolymerizing enzymes is also an important factor in controlling enzymatic saccharification^4^. The altered lignin content after NaOH and NH_4_OH pretreatments, and lignin as well as content after H_2_SO_4_ pretreatments improved the amenability of the biomass to its enzymatic saccharification process. Results of the saccharification studies exhibited the extent at which the employed pretreatments had modified the accessibility of plant cell wall polysaccharide components to the core commercial cellulase (Fig. 5), three individual xylanases and pectinases from *Bacillus* strains M35, R31 and J208 (Supplementary Figs 4 and 5).

After application of core cellulase, raw untreated SCB biomass yielded only ~1.9% saccharification which substantially increased to 14.8–38.8% after NaOH pretreatment, 14.6–25.6% after H_2_SO_4_ pretreatment and 4.8–9.8% after NH_4_OH pretreatment (Fig. 5). Out of nine different combinations, cellulase yielded highest saccharification of 38.8% and 25.6% from 1.25% SCB loading pretreated with 1% NaOH and 1% H_2_SO_4_ respectively. Any further increase in biomass or chemical loading, during pretreatments, decreased the saccharification. Similarly, during comparison of nine AFEX pretreatment combinations, 2.5% SCB loading pretreated with 3% NH_4_OH yielded highest saccharification of 9.8% by cellulase. The saccharification increased with enhanced loading of NH_4_OH. Whereas, it initially increased and then decreased with enhanced loading of biomass. This difference of saccharification between 1.25 and 2.5% SCB loading as well as between 2.5 and 5.0% SCB loading was significant in case of all three pretreatments (*p* < 0.001). Though in literature, increased accessibility to cellulase was majorly focussed for pretreatment efficiency evaluation purpose, detailed results are provided for individual xylanase (Supplementary Fig. 4) and pectinase (Supplementary Fig. 5). The major outcomes from individual enzyme mediated saccharification are listed and compared with available report in Table 2. During saccharification by individual core and accessory enzymes, maximum saccharification was observed for cellulase which decreased for xylanase and pectinase. This can be attributed to the cellulose, hemicellulose and pectin content present in biomass. After NaOH, H_2_SO_4_ and NH_4_OH pretreatments, the respective increase of ~8–20, ~7–13 and ~4–8 fold in saccharification by cellulase suggests the enhanced accessibility of structural cellulose to commercial cellulase. Similarly, these pretreatments increased M35, R31 and J208 xylanase and M35, R31 and J208 pectinase mediated saccharification by ~2–6 fold. The highest saccharification from NaOH can be attributed to the removal of major lignin fraction during NaOH pretreatment. Whereas, removal of acid soluble lignin and hemicellulose has increased saccharification, but presence of insoluble lignin fraction has kept it restricted to a moderate level. Partial removal of lignin fraction during NH_4_OH pretreatment increased biomass accessibility to lesser extent. This increase was significant for each pretreatment combinations except for 1.25% SCB loading treated with 1% NH_4_OH (*p* < 0.01, significance not marked). When compared for individual biomass loading, the difference of saccharification between 1.25 and 2.5% SCB loading as well as 2.5 and 5.0% SCB loading was more significant for NaOH and H_2_SO_4_ and NH_4_OH pretreatments in case of all three xylanases and for NaOH and H_2_SO_4_ only in case of all three pectinases (*p* < 0.001).

**Table 2:** % Saccharification observed after application of commercial core cellulase and individual accessory xylanase and pectinase enzymes.

	Biomass	Pretreatment	Individual enzymes/Combination	Saccharification/Conversion	Reference
Commercial core cellulases	Napier grass	NH_4_OH	Cellulase and Cellobiase	~60%	^50^
	Sugarcane bagasse	Microwave	Commercial cellulase (Zytex)	~66%	^36^
	Cotton stalks	NaOH	Celluclast 1.5 L and Novozyme 188	~38%	^51^
	*L. camara*, *P. juliflora*, and Corn cob	Alkali	Celluclast 1.5 L and Novozyme 188	50.8–55.0%	^52^
		Acid		39.2–48.0%	
		Chlorite		86.4–91%	
	Wheat straw	Ionic Liquid	Celluclast 1.5 L and Novozyme 188	45.9%	^34^
	Switchgrass	Alkali	Celluclast 1.5 L and Novozyme 188	~38%	^53^
	Sugarcane bagasse	Raw	Primafast 200	1.93%	This study
		NaOH		22.75–38.84%	
		Acid		14.58–25.56%	
		Liquid ammonia		6.53–9.61%	
Accessory xylanase and pectinase enzymes	Sorghum straw	Untreated	Crude xylanase from *B. altitudinis* DHN8	0.4%	^54^
		Acid		2.0%	
		Alkali		2.5%	
		Alkali peroxide		3.5%	
	Brewer’s spent grain	Aqueous ammonia	Partially purified xylanase from *B. amyloliquefaciens* XR44A	~43%	^55^
	Wheat straw	Untreated	Crude xylanase from *Bacillus* sp. CX6	~6%	^56^
	*Sugarcane Bagasse*	Alkali	Crude xylanase from *B. safensis* M35	15.6%	This study
			Crude xylanase from *B. altitudinis* R31	13.6%	
			Crude xylanase from *B. altitudinis* J208	12.9%	
			Crude pectinase from *B. safensis* M35	5.8%	
			Crude pectinase from *B. altitudinis* R31	5.7%	
			Crude pectinase from *B. altitudinis* J208	5.6%	

In literature, plenty of reports are available showing saccharification studies of variedly pretreated agro-waste biomass after subjecting them to either core commercial cellulase or accessory enzymes and diverse combinations of theirs. Few of them are listed and compared with the results in present studies (Table 2). 2.5% SCB (pretreated with 1% H_2_SO_4_ followed by range of 0.5–5.0% NaOH) with loading of 25 FP units of Accellerase 1500, 73% cellulose digestibility was observed after 48 h, whereas in present studies use of only ~6.5 FP units on 2% SCB loadings pretreated with only NaOH at different biomass and PSCB loadings, exhibited 22–38% cellulose digestibility in terms of saccharification after 60 h^16^. Several other comparable reports have been published by investigators using commercial cellulase Cellic® Ctec3 on steam exploded SCB and using mixture of Spezyme CP and Novozyme 188, containing 30 units of each, on ammonia treated SCB^19,38^. In extensive studies on enzymatic saccharification of polysaccharides from different PSCB at 2% SCB biomass loading with individual loading of 6 mg of commercial cellulase (1:4 mixture of Celluclast 1.5L and Novozyme 188), endo-xylanase and pectinase per g of biomass, amount of sugars released by pectinase, xylanase and cellulase was in increasing order. Further, NaOH, steam explosion, H_2_SO_4_ and H_2_O_2_ was the order in which the saccharification decreased. These observations are in concurrence with the results of presented saccharification studies^39,40^.

### Light and fluoresce based microscopic studies of sugarcane biomass

**Figure 6:**
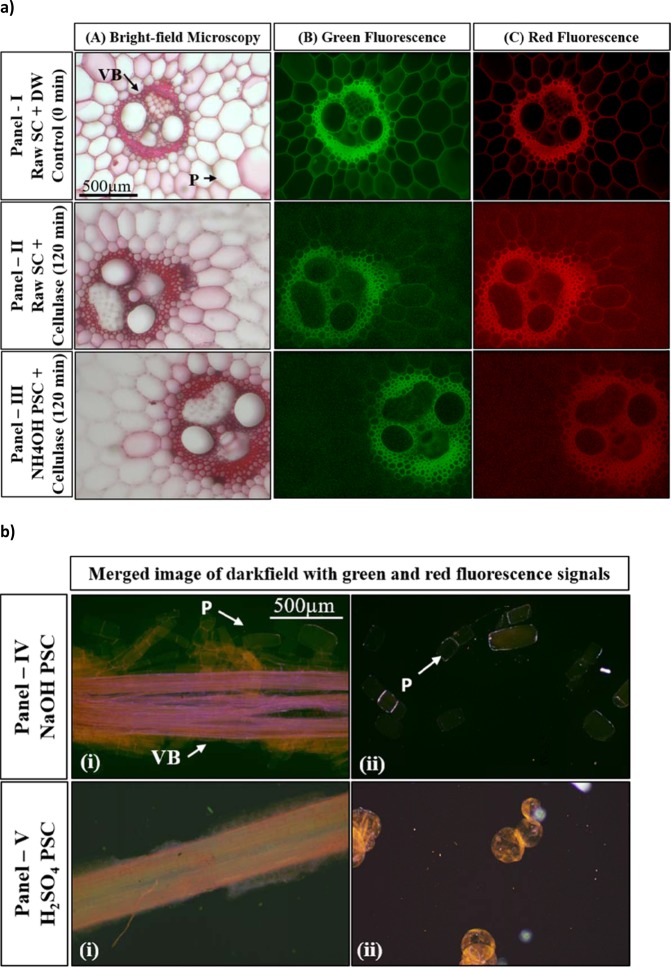
Brightfield and fluorescence microscopy analysis of safranin stained pretreated Sugarcane (SC) biomass. (**a**) Panel-I-III: Brightfield and fluorescence microscopy of safranin stained raw and NH_4_OH pretreated SC transverse sections before and after incubation with cellulase: Panel I- Raw SC before enzyme treatment, Panel II- Raw SC and Panel III- NH_4_OH pretreated SC after 120 min of cellulase treatment; Images captured for Brightfield micrograph (Column A), Green fluorescence signal (Column B) and Red fluorescence signal (Column C); Images are taken at 10X magnification; (**b**) Brightfield microscopy of safranin stained NaOH treated (Panel IV) and H_2_SO_4_ pretreated (Panel V) sugarcane biomass fraction: (i) Fibrous vascular bundle (labelled as FVB) and the fragments of surrounding parenchymatous pith cells; (ii) Individual cells of pith parenchyma separated after NaOH and H_2_SO_4_ pretreatments (All images at 10X magnification).

The structural and anatomical details for SCB stem is provided in Supplementary Fig. 6 ^41^. Anatomical changes brought in to sugarcane cuboids due to pretreatments and their effects on the cellulase mediated breakdown of cellulosic cell wall components from safranin stained pretreated sugarcane (PSC) transverse sections (TS) were microscopically visualized after focusing the stele region, comprising vascular bundle (VB) of xylem-phloem embedded in medullar pith of parenchymatous (P) tissue. Safranin staining imparted red colour to the cell wall under brightfield microscopy (Supplementary Fig. 7). Whereas, it yielded green fluorescence signal from cellulosic components and red fluorescence signal from lignin components present in the cell wall during fluorescent microscopy. Merged fluorescence of green and red in an overlapping image suggested the close association and co-distribution of cellulose and lignin together, in the most parenchymatous cell wall (blue arrow, Supplementary Fig. 7). In case of sclerenchyma bundle sheath, the development of secondary cell wall containing co-deposition of cellulose and lignin increases the thickness of the cells wall. In some innermost cells of Sclerenchyma tissue and Phloem, there is clear green fluorescence suggesting the less lignin and more cellulosic content (white dotted arrow, Supplementary Fig. 7). A similar observations have been reported for the sugarcane stem^41^. Such intact cells with clear cell wall can be seen for untreated raw sugarcane plant sections from safranin stained bright field image and green and red fluorescence image (Fig. 6a, Panel-I). Cellulase treatment of 120 min to the safranin stained TS of raw and NH_4_OH PSC stele exhibited the discontinuous appearance of the cell wall from parenchymatous pith tissue in brightfield micrographs and reduced intensity of green fluorescence in the fluorescence micrographs (Fig. 6a, Panel-II and III). Though, the pattern of cellulase action was more or less similar, the several layers of pith tissues closely surrounding the FVB, which were found intact in raw biomass (Panel-II) were noticeably ruptured in NH_4_OH PSC biomass (Panel-III). Such increased disruption of the inner most layers of parenchymatous cells surrounding the vascular bundles can clearly be attributed to the increased accessibility of cellulose to the cellulase in NH_4_OH pretreated biomass due to partial removal of lignin from the cell wall matrix which was inaccessible in raw untreated biomass. Moreover, the sieve plates and companion cells, the two major components of phloem vasculature from VB, also exhibited disappearance of green fluorescence, at the same time the lignin rich xylem components and the sclerenchyma sheath of the vascular bundle pursued red fluorescence (Fig. 6a, Panel-II and III).

The NaOH and H_2_SO_4_ pretreatments shattered stele of sugarcane biomass into FVB and parenchymatous pith cells (Fig. 6b). Microscopic examination of safranin stained fibrous filaments under light microscopy revealed that the parenchymatous pith region had completely disintegrated into either individual cells or small chains of cells leaving vascular bundles apart. This made the sectioning of stele and its enzymatic hydrolysis difficult to perform under microscopy. The possible reason behind the shattering of the vascular bundles from parenchyma pith biomass might be attributed to the removal of large amount of lignin and other soluble components, distributed at middle lamellae and corners of the plant cell wall. These observations corroborate over earlier observations of gravimetric, HPLC, FTIR and SEM analyses. Moreover, in case of sugarcane biomass, the lignin distribution in middle lamella, of two cells, corner of cells and in secondary cell wall have been shown using fluorescence and transmission electron microscopy and ultrastructure cytochemistry techniques^41,42^. Removal of such lignin must have freed the cells and enhanced area of the surfaces to increase the accessibility of cellulose and hemicelluloses to their respective saccharifying enzymes as it was seen for cellulase, xylanase and pectinase mediated saccharification. Microscopy of individual parenchyma cells unveiled the intact rectangular shape of cells maintained after NaOH pretreatments, which was drastically altered to swollen spherical shape after H_2_SO_4_ pretreatment (Fig. 6b, Panel- IV and V). The overlap of darkfield, and fluorescence images for these vascular bundles and individual cells, explains such drastic differences between the cell morphology (Fig. 6b; Supplementary Fig. 8). Removal of hemicellulose components along with lignin during H_2_SO_4_ pretreatment must have disturbed the integrity of the cell wall matrix making the cell surface more wrinkled and uneven. The removal of lignin but not hemicellulosic fractions after NaOH pretreatment have separated the cells and maintained their structures intact. This resulted in increased biomass accessibility to cell wall degrading enzymes making biomass amenable for saccharification and valorizing it.

In conclusion, the effect of NaOH, NH_4_OH and H_2_SO_4_ pretreatment on structural polysaccharide digestibility of SCB were studied. After cellulase amendment, highest (38.8%) saccharification was obtained from NaOH PSCB followed by 25.56% and 9.61% from H_2_SO_4_ and NH_4_OH PSCB. Biochemical, HPLC, gravimetric, FTIR, SEM and microscopic analyses supported these observations by revealing several features of these pretreatments. NaOH pretreatment efficiently removed lignin without affecting cellulosic and hemicellulosic content. H_2_SO_4_ pretreatment efficiently removed hemicellulosic components and only acid soluble lignins disturbing the structural integrity of cell wall matrix. These, effects resulted in separation of individual parenchymatous pith cells from the fibrous vascular bundles. Similarly, NH_4_OH resulted in partial delignification and negligible alteration in polysaccharide components while damaging pith regions from SCB. These effects increased structural polysaccharide accessibility to PCWD enzymes and enhanced saccharification. Though, due to restriction of unremoved lignin, the saccharification in case of H_2_SO_4_ and NH_4_OH PSCB was lower compared to NaOH PSCB. Thus, the SCB biomass is valorized as its amenability for the enzymatic saccharification has been increased. This work opens up a window for evaluation of the different commercial as well as bacterial enzymes for their application in biomass saccharification after NaOH pretreatments.

## Materials and Methods

### SCB biomass preparation for pretreatment

Wet SCB, collected from local market, was comminuted in a grinder and washed with hot (~60 °C) distilled water (DW) in shaking conditions at 120 rpm up to 1 h and separated by filtration to remove soluble sugars from ground biomass. The process was repeated till the filtrate showed no reducing sugar. This ground, washed bagasse was heat dried in a hot air oven at 60 °C till the weight remained constant for three subsequent measurements at one-hour interval. The fractions that ranged between 0.5–5.0 mm were sieved and separated from dried-ground SCB and stored in airtight container at room temperature^43^. SCB biomass was subsequently subjected to chemical pretreatments and enzymatic saccharification studies. All chemicals required for studies were of analytical grade and purchased from HiMedia (Mumbai, India) or Sigma-Aldrich (Missouri, USA).

### Pretreatment of SCB biomass

Alkali, liquid ammonia and acid pretreatments were individually carried out in the autoclave vessel with loading capacity of 15.5 L. SCB biomass was individually pretreated with NaOH, NH_4_OH and H_2_SO_4_. Combinations of three different concentrations of each chemical agent (1.0, 2.0 and 3.0%) and three different concentrations of SCB loadings (1.25, 2.50 and 5.0%) were performed individually in 250 ml Erlenmeyer flasks. Heat, pressure and time were the physical parameters maintained constant to all pretreatments. Pretreatments were performed at holding time of 20 min at 10 psi (115 °C) and then the pressure was released gradually. Schematic representation of the pretreatment strategy is provided in Supplementary Fig. 1. Biomass soaked in 100 ml DW at room temperature and pressure for 20 min without any chemical pretreatment was used as control and referred to as raw or untreated biomass control. After pretreatments, all samples were cooled at room temperature and filtered through the nylon sieve filter. Centrifugation at 10,000 rpm for 20 min was performed to separate the remaining filtrate from biomass. Thus, separately collected filtrate and pretreated sugarcane bagasse (PSCB) biomass were subjected to following analyses.

### Estimation of released SRS from filtrate

Released soluble reducing sugar (SRS) in filtrate was quantified using 2,4- dinitro salicylic acid (DNS) reagent method^44^. 300 μl of DNS solution was added to 300 μl of sampled filtrate aliquot. The mixture was boiled for 10 min. Volume was made up to 1.5 ml after cooling and A_540nm_ was measured. The amount of released SRS in filtrate during pretreatment from SCB was quantified using D-glucose as standard and expressed as % released SRS for further comparisons as mentioned below.

1 $$ \% \,released\,SRS=\frac{reducing\,sugar\,released\,in\,filtrate\,during\,pretreatment\,(mg)}{biomass\,used\,for\,pretreatment\,(mg)}\times 100$$

### HPLC analysis of filtrate for monomeric sugars

20 µl of aliquot from each pretreatment filtrate was loaded in Hi-Plex-H column (containing the stationary phase of strong cation exchanger resin made up of sulfonated, cross-linked styrene-divinylbenzene copolymer in hydrogen form, Agilent Technologies, USA) on HPLC system (Shimadzu, Japan) equipped with LC-10AT pump, CTO-10ASVP oven column cabinet and detected using RID-10A detector. D-glucose, and D-xylose were used as standards. Soluble products were separated at 60 °C under isocratic conditions using deionized water as mobile phase at flow rate of 0.6 ml min^−1^ as recommended by Agilent technologies. Chromatographs for individual samples and standard controls were compared with respect to retention time (RT) to identify the released monomers.

### Gravimetric analysis of PSCB biomass

After filtration and centrifugation, collected solid PSCB biomass was washed several times with fresh DW to neutralize the pH, squeezed, and images were taken using Sony DSC-WX150 Point & Shoot Camera (Supplementary Fig. 2). After recording visible morphology, untreated and pretreated biomass was collected in pre-weighed Petri dishes, samples were heat dried in a hot air oven at 60 °C till the weight remained constant for three subsequent measurements at one-hour interval. The dried biomass was stored in air tight container at room temperature and used for further analysis. Decrease in weight of dry biomass was calculated as mentioned below and expressed as % weight loss in biomass.

2 $$ \% \,weight\,loss\,in\,biomass=\frac{biomass\,obtained\,after\,drying\,(mg)}{biomass\,used\,for\,pretreatment\,(mg)}\times 100$$

Further, amount of soluble components, other than SRS, released due to pretreatment from biomass was also calculated using the following formula.

3 $$ \% \,Other\,soluble\,components=( \% \,Weight\,loss\,in\,biomass- \% \,Released\,SRS)\,$$

### FTIR analysis of SCB biomass

Solid SCB samples were sent to FTIR lab at Central Research Facility (CRF, IIT-Kharagpur, West Bengal, India). After mixing with potassium bromide (KBr), the samples were pressed to form a thin disc. FTIR spectra were obtained over the range of 400–4000 cm^−1^ with a spectral resolution of 0.5 cm^−1^ wavenumber^32,45^. The data was analyzed using the reference peaks available in literature for chemical changes in the structural components of lignocellulosic biomass^33,34,46,47^.

### SEM analysis of PSCB biomass

All heat dried solid SCB samples were collected in a microfuge tube and washed with phosphate buffered saline (PBS) pH 7.2 ± 0.2, fixed in 2.5% v/v glutaraldehyde for 15 min, again washed with PBS and dehydrated in a series of increasing acetone concentrations i.e., 10, 25, 50 and 75% for 10 min each and stored in 100% absolute acetone at −20 °C till further analysis^48^. For imaging process, air dried sample was placed on a prefixed adhesive carbon tape on metal stub and sputter coated with Platinum (Pt) in Auto Fine Coater (JEOL-JFC-1600). Sample were examined at 10 kV under SEM (JEOL, JSM-7600F-FEG-SEM) at Sophisticated Analytical Instrument Facility (SAIF, IIT-Powai, Mumbai, India). Electron micrographs were taken at desired magnifications and analyzed for structural changes in morphology at cellular level.

### Enzyme preparations for biomass saccharification

Three lab strains, viz., *B. safensis* M35, *B. altitudinis* R31 and *B. altitudinis* J208, were used as source of xylanase and pectinase enzymes. Preparation of cell free supernatant as crude source of xylanase and pectinase was done by growing these isolates in production medium BHM-YEP amended with Wheat bran (WB) and Citrus peel (CP) respectively. BHM-YEP contained (%w/v) Bushnell Haas Medium 0.327%, Yeast Extract 0.025%, and peptone 0.075%. After 48 h of incubation, the liquid medium was centrifuged at 10,000 rpm and protein content as well as enzyme activities were quantified as mentioned in our earlier studies^22,23^. Specific activities (units/mg protein) were calculated to be 144.2, 136.3 and 139.6 units for M35, R31 and J208 xylanases; 2.0 * 10^5^, 1.9 * 10^5^ and 2.1 * 10^5^ units for M35, R31 and J208 pectinases. Crude cell free enzyme of core cellulase Primafast 200 (Genencor, Du-Pont) with specific activity of 7.56 units/mg protein was commercially procured. These cell free supernatants were used as individual crude enzymes to study the amenability of raw and pretreated SCB biomass for enzymatic saccharification.

### Microscopic analysis of pretreated sugarcane biomass anatomy during cellulase mediated hydrolysis

In a separate experiment, 0.5 × 0.5 × 1.0 cm^3^ sized cuboid pieces were cut from the stele of fresh sugarcane stem and washed thoroughly with DW till removal of soluble sugars ceased. Two of these pieces cumulatively weighing ~1.25 g were subjected to the different pretreatments as mentioned in previous section. These pretreated sugarcane pieces were washed thoroughly with DW to neutralize their pH. Thin hand-cut transverse sections (TS) from the untreated and pretreated cuboid stems were selected for further studies. The sections were stained with 0.1% Safranin-O (prepared in 1% Ethanol), washed with water at 30 °C, mounted on clean glass slides with the 100 µl of properly diluted (0.38 units) commercial cellulase and subjected to brightfield and fluorescence based anatomical studies to understand the pretreatment effect on plant cell wall digestibility by commercial cellulase^41,49^. Brightfield and fluorescence microscopic observation of this sections on Olympus CX-41 system microscope was performed with help of CX-RFL-2 reflected fluorescence attachment. Samples were excited using the excitation filter BP475 (475 nm, blue colour range) and emission were observed in range of green fluorescence (500–515 nm) and orange-red fluorescence (570–590 nm). The images captured at 0 & 120 min were analyzed using CellSens Standard software V 2.3 (Olympus) and compared for breakdown of cell wall in various region of the TS.

### Raw and pretreated SCB biomass saccharification

2.0% w/v of biomass was suspended in 10 ml of 50 mM Tris-Cl buffer pH 7.0 and to this system 100 µg/ml of each, sodium azide, ampicillin, kanamycin and streptomycin was amended to prevent microbial contaminations. This system was amended with individual enzymes as follows. 800 µg (in terms of protein) of cellulase (C) comprising 6.05 activity units was loaded. 300 µg (in terms of protein) of each of six accessory enzymes individually from *Bacillus* strains i.e. M35, R31 and J208 xylanase comprising 43.26, 40.89 and 41.88 activity units respectively) and/or M35, R31 and J208 pectinase (comprising 6.00*10^4^, 5.70*10^4^ and 6.30*10^4^ activity units respectively) was amended. This system was incubated in shaking condition at 160 rpm, 40 °C up to 60 h. Hydrolysate samples were collected at 60 h and analyzed for saccharification through DNS method. The % saccharification values are calculated as follows^31^.

4 $$ \% \,saccharification=\frac{reducing\,sugar\,released\,by\,enzymatic\,hydrolysis\,(mg)}{initial\,solid\,biomass\,used\,for\,hydrolysis\,(mg)}\times 100$$

## Supplementary information

Supplementary figure.
