# Supplementary material for: Valorization of sugarcane bagasse by chemical pretreatment and enzyme mediated deconstruction
Source: Sci Rep. 2019 Nov 4;9:15904. doi: 10.1038/s41598-019-52347-7 (PMC6828687; doi:10.1038/s41598-019-52347-7)

Electronic Supplementary Material

Title:

Valorization of sugarcane bagasse by chemical pretreatment and enzyme mediated deconstruction

Authors:

Vihang S. Thite [a] and Anuradha S. Nerurkar [a, *]

Affiliation:

[a] Department of Microbiology and Biotechnology Centre, Faculty of Science. The Maharaja Sayajirao University of Baroda, Vadodara, Gujarat, India. 390002.

E-mail and ORCID-ID:

Vihang S. Thite: thitevihang87@yahoo.com;

ORCID-ID: 0000-0003-4197-4656

Anuradha S. Nerurkar*: anuner26@yahoo.com;

ORCID-ID: 0000-0002-6674-4032

*** Correspondence:**

Prof. Anuradha S. Nerurkar

Department of Microbiology and Biotechnology Centre, Faculty of Science. The Maharaja Sayajirao University of Baroda, Vadodara, Gujarat, India. 390002.

Supplementary Figure 1: Schematic of the pretreatment strategy employed under the presented study:

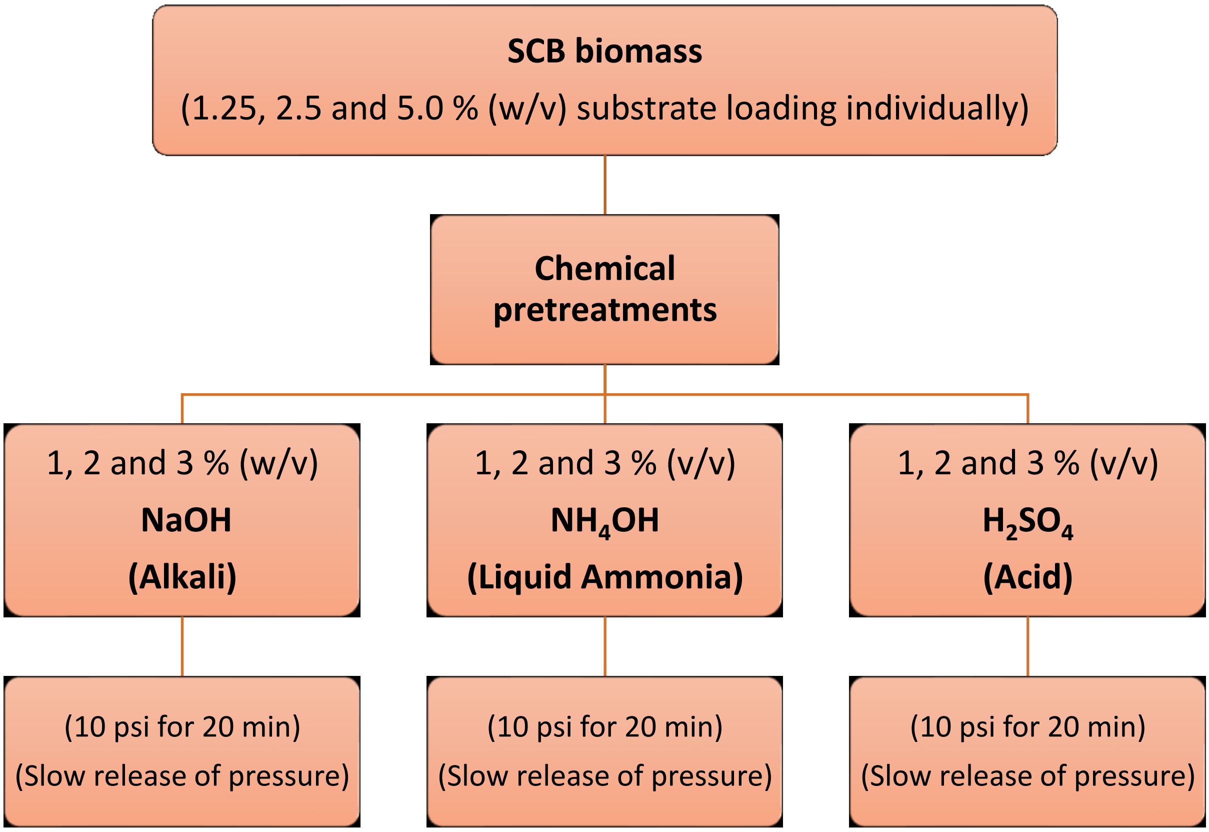

Supplementary Figure 2: Visible morphological differences in raw and PSCB biomass. Representative images (taken using Sony DSC-WX150 Point & Shoot Camera) showing morphological appearance of ground and fractionated SCB biomass: **(a)** Raw-untreated SCB; and SCB pretreated with **(b)** Alkali (NaOH), **(c)** Liquid ammonia (NH_4OH) and **(d)** Acid (H_2SO_4) methods at 5.0% SCB loading and 3.0% chemical loading; **(e)**: Morphological appearance of intact pith cuboids from SCB stem before and after pretreatments of 1.25% SCB loading and 1.0% chemical loading.

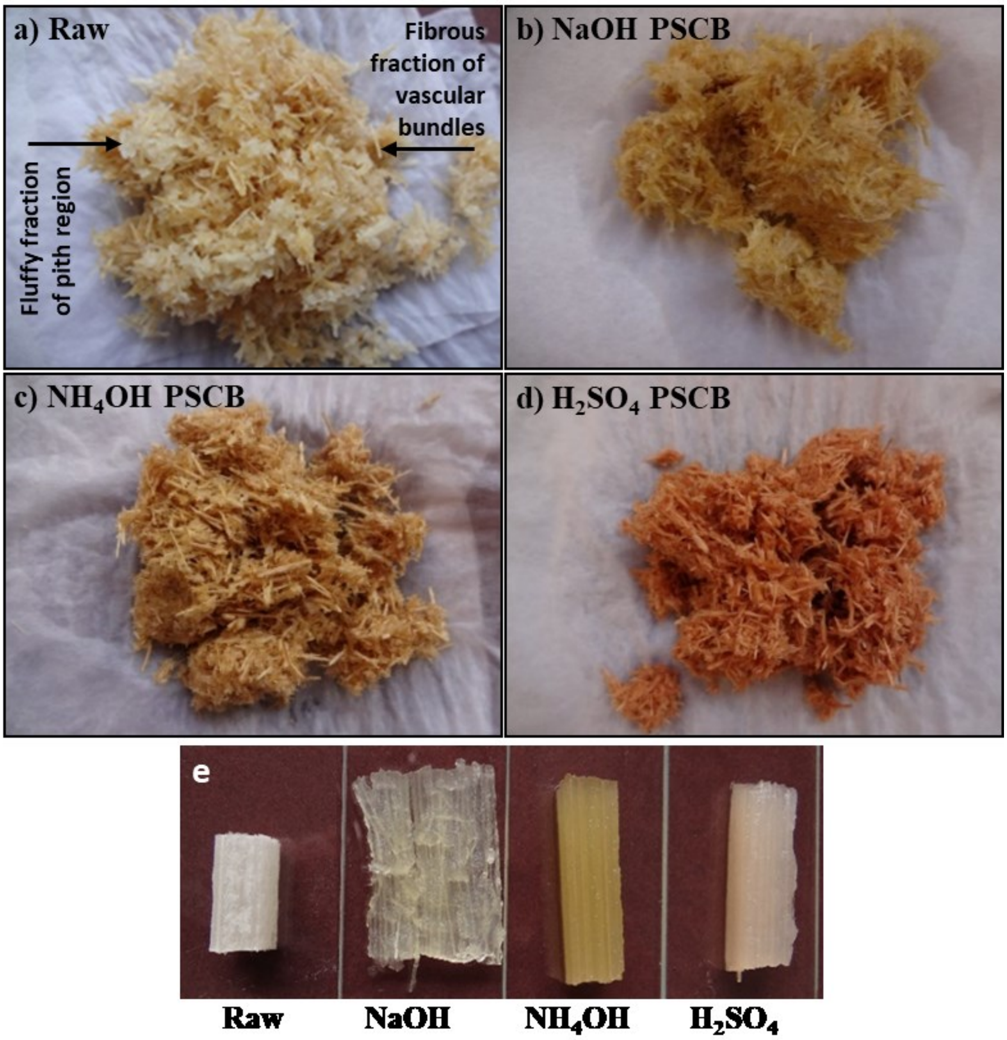

Supplementary Figure 3: Contour plots representing release of reducing sugar and dry weight loss at different loading values of biomass and chemicals: (A-C) Left Panel: % released soluble reducing sugar due to **(A)** NaOH, **(B)** NH_4OH and **(C)** H_2SO_4 pretreatment; **(D-F) Right panel:** % loss in dry weight of biomass due to **(D)** NaOH, **(E)** NH_4OH and **(F)** H_2SO_4 pretreatment.

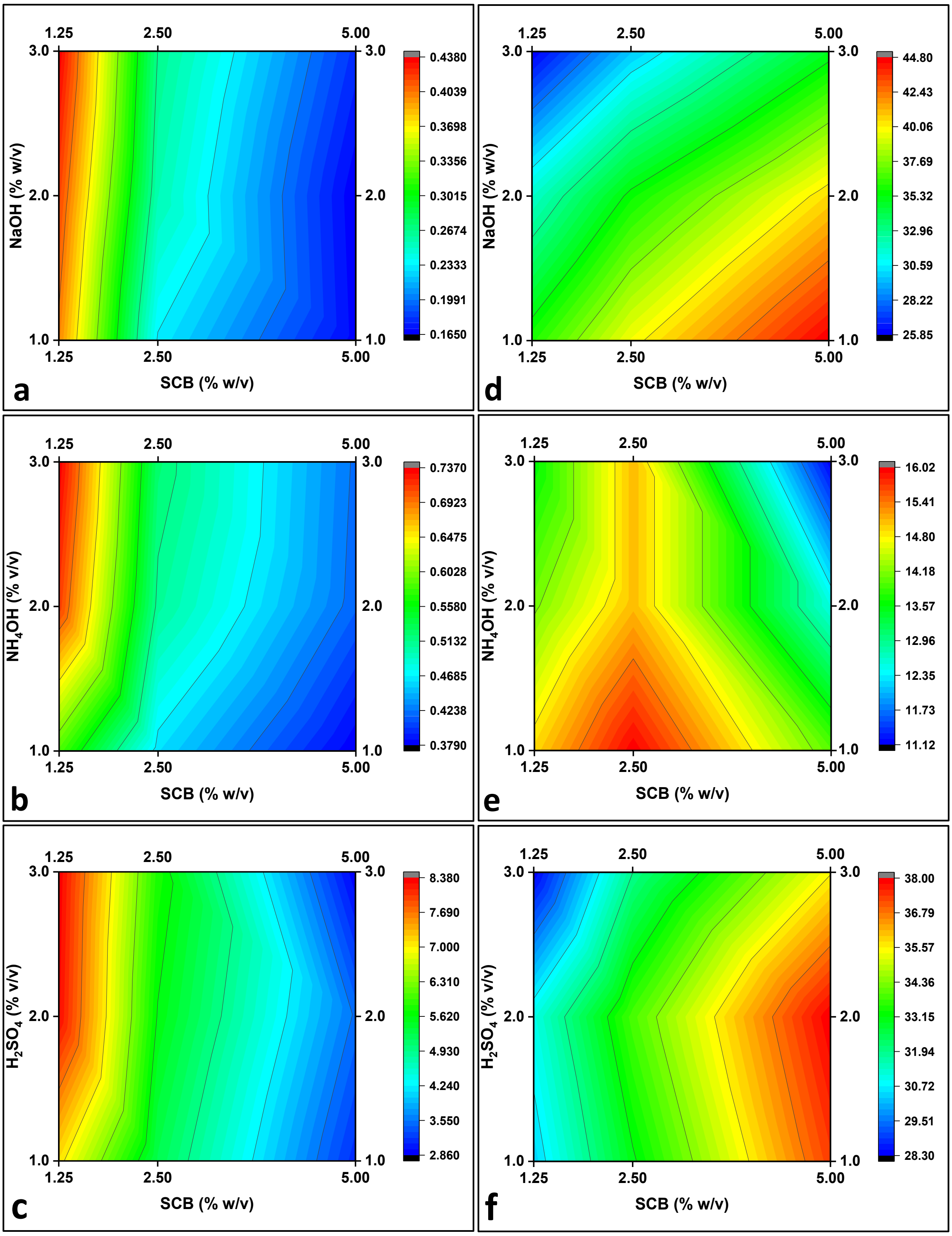

Based on combinations of the biomass and chemical loadings, varied patterns for % released SRS and % dry weight loss for all three pretreatment methods, were observed in the contour plots (Supplementary Figure 3 a-f). NaOH, NH_4OH and H_2SO_4 was the pretreatment order in which overall release of SRS increased. This release of SRS followed a similar pattern for all three pretreatments, i.e., it increased with enhanced chemical reagent loadings and decreased with enhanced biomass loadings (Supplementary Figure 3 a-c). NH_4OH, H_2SO_4 and NaOH was the pretreatment order in which overall % dry weight loss of SCB increased yet the pattern was drastically different for each chemical pretreatment (Supplementary Figure 3 d-f). During alkali pretreatment weight loss increased with enhanced NaOH loadings and decreased with enhanced biomass loadings (Supplementary Figure 3d). During AFEX pretreatment weight loss decreased with enhanced NH_4OH loadings whereas, after initial increase, it decreased with enhanced biomass loading (Supplementary Figure 3e). During acid pretreatment, weight loss increased with enhanced H_2SO_4 loadings. Weight loss increased initially and then decreased with enhanced biomass loading (Supplementary Figure 3f).

Supplementary Figure 4: Xylan accessibility from raw and PSCB by crude xylanase enzymes. % Saccharification of raw and PSCB by crude xylanases obtained from (**a**) *B. safensis* M35, (**b**) *B. altitudinis* R31 and (**c**) *B. altitudinis* J208; Significance difference between 1.25% (□), 2.5% (□) and 5.0% (□) SCB biomass loading for pretreatments is given as * = $p < 0.05$, ** = $p < 0.01$, *** = $p < 0.001$ and **** = $p < 0.0001$ for Sidek test-Two-way ANOVA analysis; Columns and Error bars represents Mean and Standard Error of Mean (SEM) respectively for n=3.

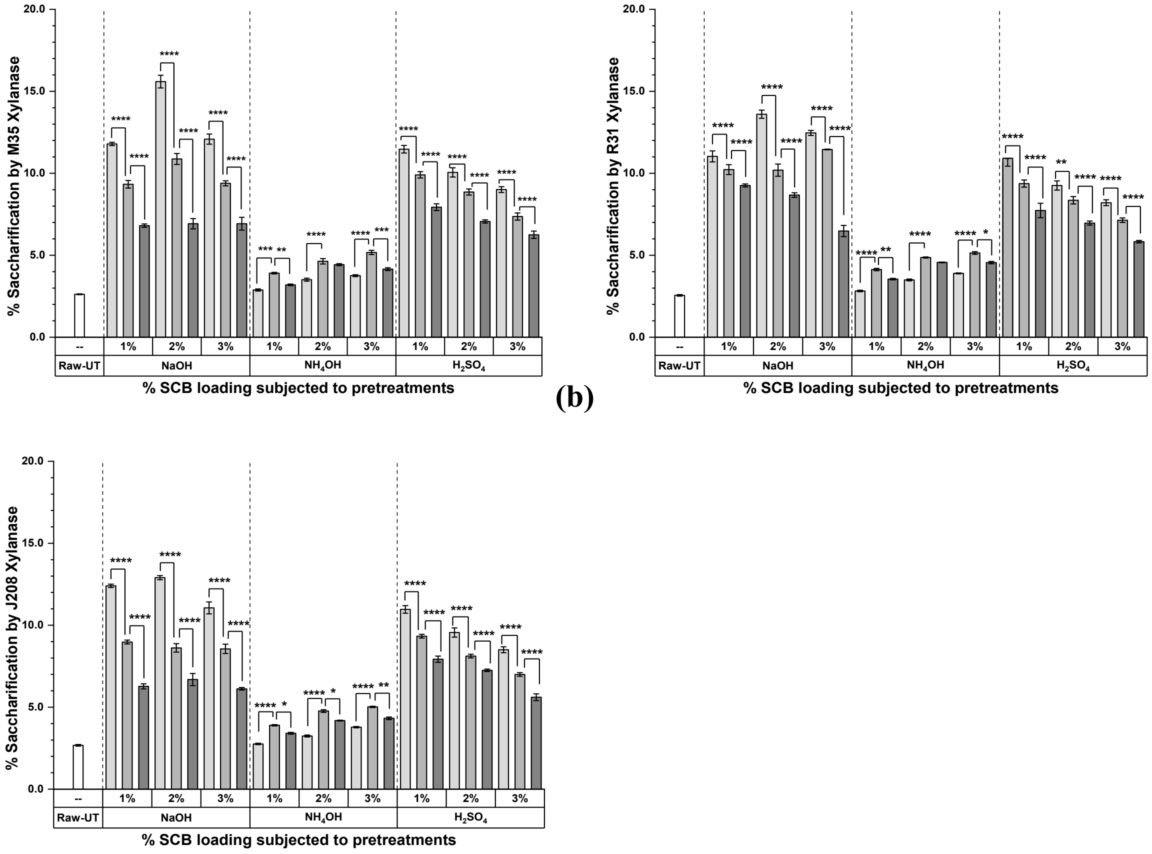

(a) (b) (c)

Supplementary Figure 5: Pectin accessibility from raw and PSCB by crude pectinase enzymes. % Saccharification of raw and PSCB by crude pectinases obtained from (**a**) *B. safensis* M35, (**b**) *B. altitudinis* R31 and (**c**) *B. altitudinis* J208; Significance difference between 1.25% (□), 2.5% (□) and 5.0% (□) SCB biomass loading for pretreatments is given as * = $p < 0.05$, ** = $p < 0.01$, *** = $p < 0.001$ and **** = $p < 0.0001$ for Sidek test-Two-way ANOVA analysis; Columns and Error bars represents Mean and Standard Error of Mean (SEM) respectively for n=3.

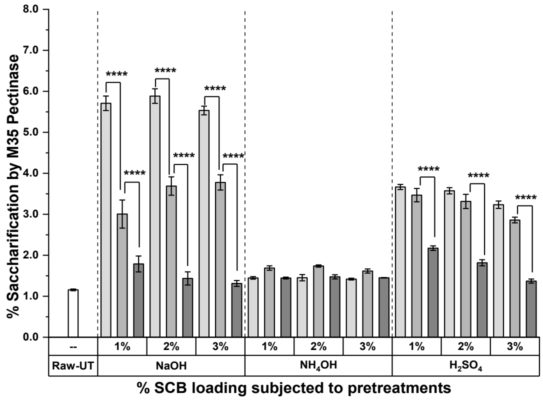

(a)

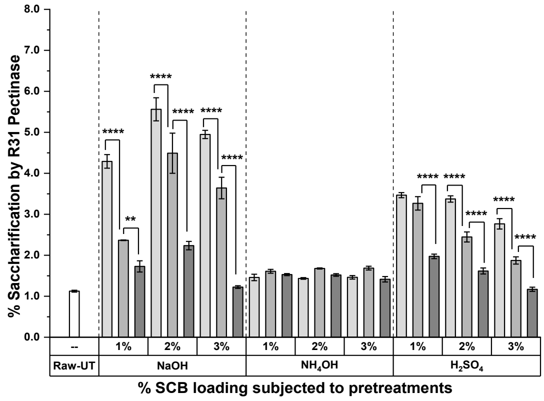

(b)

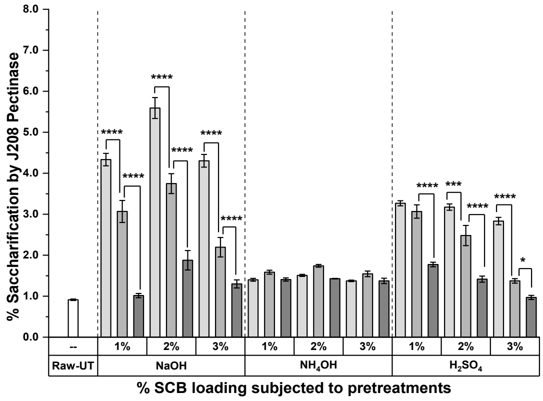

(c)

Supplementary Figure 6: Anatomy of vascular bundle in sugarcane stem section: Transverse section of sugarcane stem prepared in this work representing a single vascular bundle observed after safranin staining at 10X magnification under brightfield microscope.

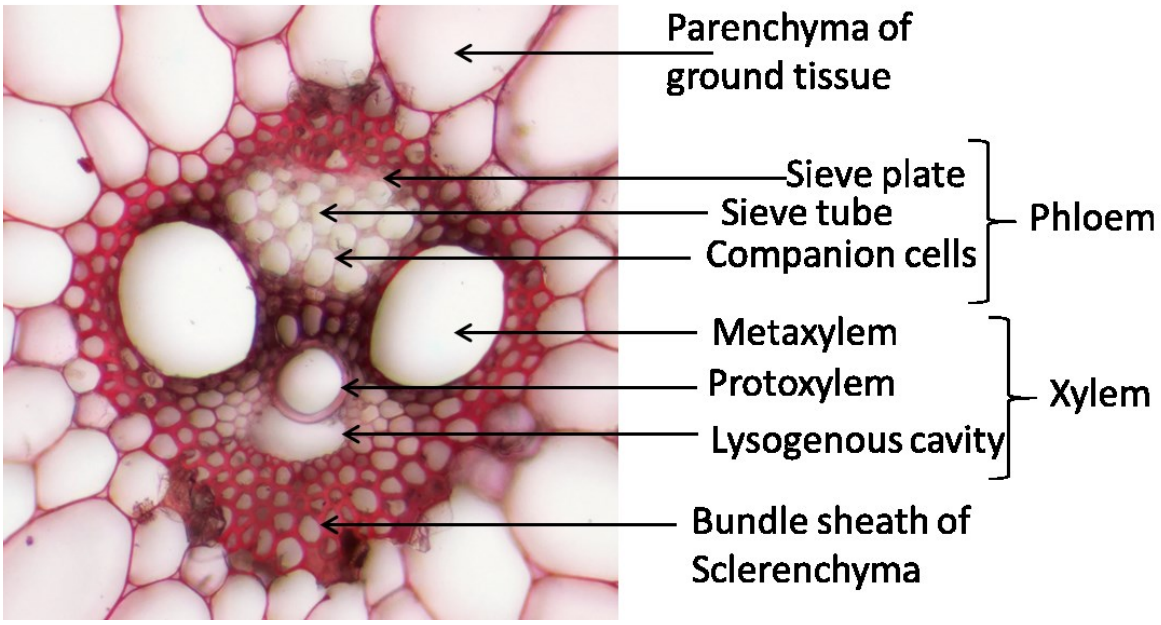

Bundle sheath comprises of two to several layers of sclerenchyma tissue cells which surrounds each vascular bundle. The vascular bundle mainly composed of xylem and phloem. The xylem vessels consist of two different type of cavity cells. The vascular bundles in sugarcane bagasse are collateral type where xylem vessels are arranged in the form of letter ‘Y’ or ‘V’ or ‘U’. Arms of the alphabets are formed by large metaxylem vessel elements while the rest is composed by protoxylem. The part of protoxylem disintegrates in later stages and the lysogenous or protoxylem cavity or lacuna is formed. As the phloem elements are situated in the grooves of Y or V or U-shaped xylem, the xylem encircles phloem on the three sides. Among, above mentioned tissues, protoxylem, metaxylem and sclerenchyma cells are the dead ones which contains lignin depositions on the lumen side of the cell wall. The phloem tissues lack the lignin and majorly composed of cellulosic and hemicellulosic cell wall. The parenchymatous tissues in pith region contains major cellulosic and hemicellulosic fractions in the cell wall with minor amount of polyphenolic lignin and pectin content.

Supplementary Figure 7: Visualization of cellulose and lignin content distribution in sugarcane section after safranin staining through brightfield and fluorescence microscopy.

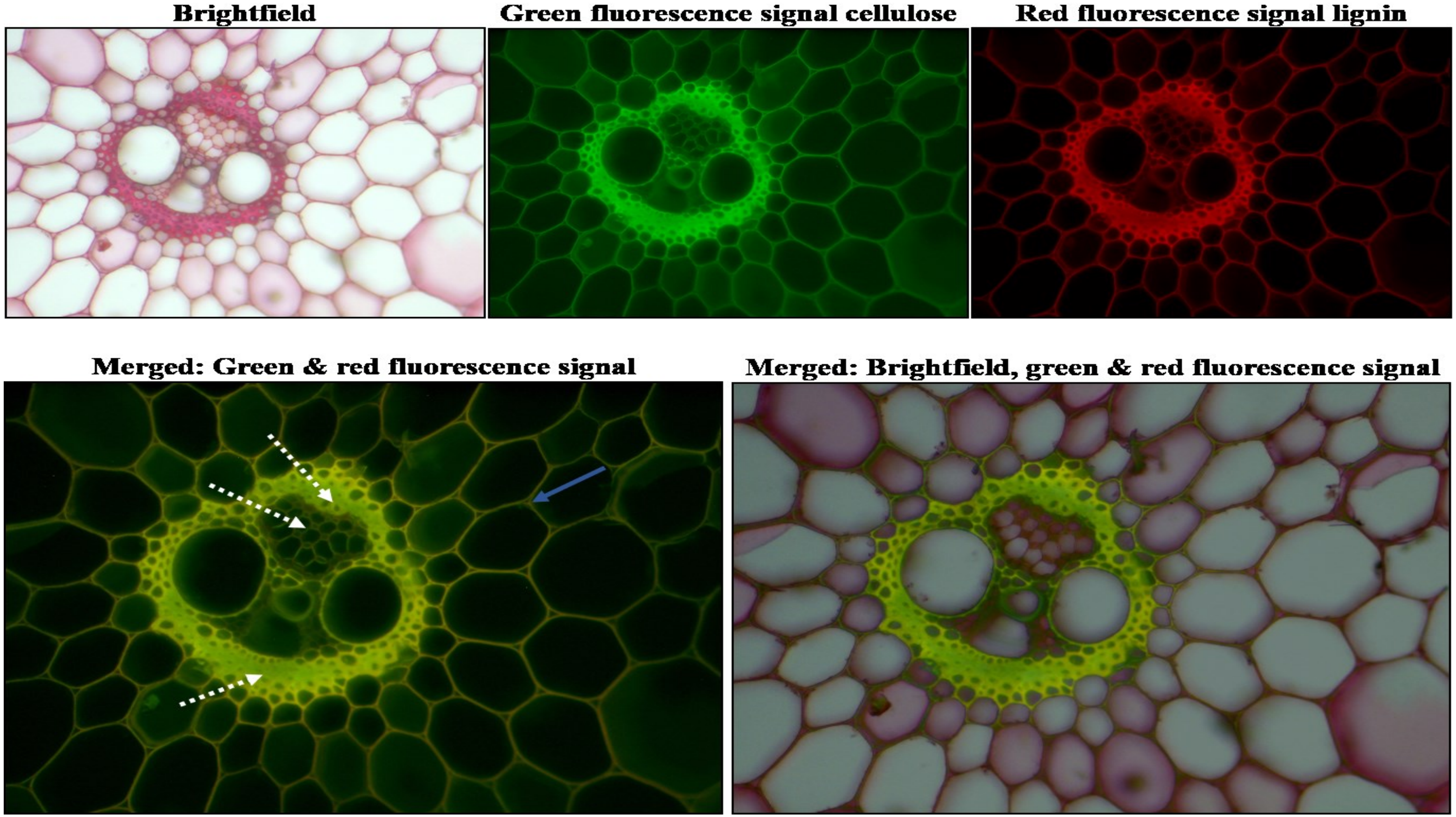

Supplementary Figure 8: Brightfield, Darkfield and Fluorescence microscopy images showing sugarcane biomass deconstruction:

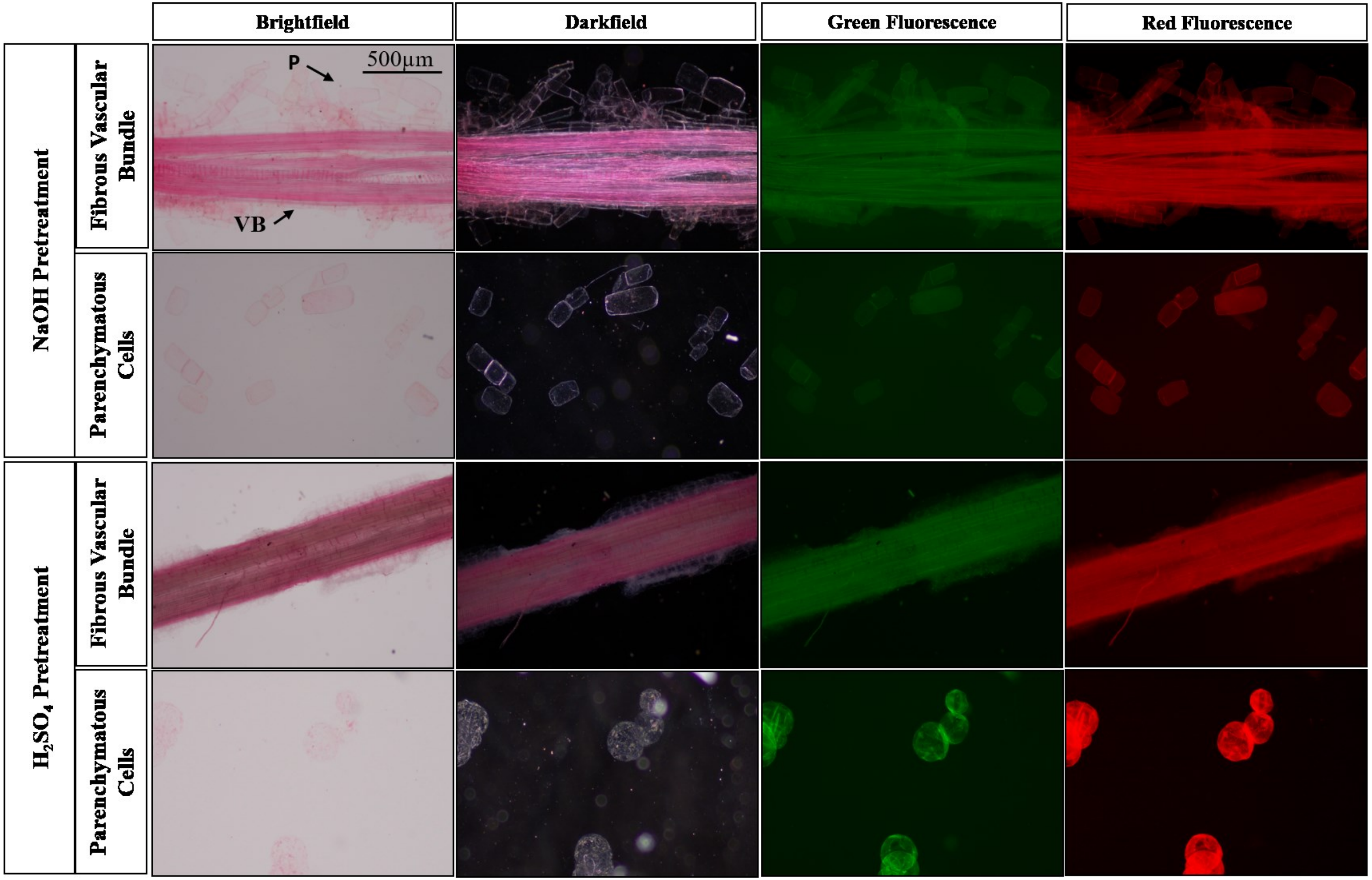

Supplement: Supplementary file 1 — Supplementary figure. [file 41598_2019_52347_MOESM1_ESM.pdf]
